# Liver Afferents Contribute to Water Drinking-Induced Sympathetic Activation in Human Subjects: A Clinical Trial

**DOI:** 10.1371/journal.pone.0025898

**Published:** 2011-10-07

**Authors:** Marcus May, Faikah Gueler, Hannelore Barg-Hock, Karl-Heinz Heiringhoff, Stefan Engeli, Karsten Heusser, André Diedrich, André Brandt, Christian P. Strassburg, Jens Tank, Fred C. G. J. Sweep, Jens Jordan

**Affiliations:** 1 Institute of Clinical Pharmacology, Medical School Hannover, Hannover, Germany; 2 Department of Nephrology, Medical School Hannover, Hannover, Germany; 3 Department for General, Abdominal, and Transplant Surgery, Medical School Hannover, Hannover, Germany; 4 Department of Medicine, Division of Clinical Pharmacology, Autonomic Dysfunction Center, Vanderbilt University School of Medicine, Nashville, Tennessee, United States of America; 5 Department of Biomedical Engineering, Vanderbilt University, Nashville, Tennessee, United States of America; 6 Department of Laboratory Medicine, Radboud University Nijmegen Medical Centre, Nijmegen, the Netherlands; 7 Department for Gastroenterology, Hepatology and Endocrinology, Medical School Hannover, Hannover, Germany; University of Sydney, Australia

## Abstract

Water drinking acutely increases sympathetic activity in human subjects. In animals, the response appears to be mediated through transient receptor potential channel TRPV4 activation on osmosensitive hepatic spinal afferents, described as osmopressor response. We hypothesized that hepatic denervation attenuates water drinking-induced sympathetic activation. We studied 20 liver transplant recipients (44±2.6 years, 1.2±0.1 years post transplant) as model of hepatic denervation and 20 kidney transplant recipients (43±2.6 years, 0.8±0.1 years post transplant) as immunosuppressive drug matched control group. Before and after 500 ml water ingestion, we obtained venous blood samples for catecholamine analysis. We also monitored brachial and finger blood pressure, ECG, and thoracic bioimpedance. Plasma norepinephrine concentration had changed by 0.01±0.07 nmol/l in liver and by 0.21±0.07 nmol/l in kidney transplant recipients (p<0.05 between groups) after 30–40 minutes of water drinking. While blood pressure and systemic vascular resistance increased in both groups, the responses tended to be attenuated in liver transplant recipients. Our findings support the idea that osmosensitive hepatic afferents are involved in water drinking-induced sympathetic activation in human subjects.

**Trial Registration:**

ClinicalTrials.gov NCT01237431

## Introduction

Water drinking acutely increases sympathetic activity in human subjects [1]–[4]. Sympathetic activation with water drinking has an immediate onset of 1–5 minutes reaching a maximum after 30–40 minutes. The response elicits a profound increase in blood pressure in patients with impaired baroreflex function [1]. Blood pressure increases moderately in older [1] and not at all in healthy young subjects [1], [2]. Similarly, water ingestion raises blood pressure in sinoaortic denervated but not in baroreflex intact mice [5]. Pharmacological sympathetic inhibition and deletion of the norepinephrine producing gene dopamine-beta-hydroxylase abolish the pressor response [1], [5]. Water drinking also increases metabolic rate [6], [7]. Studies in patients with high spinal cord injury suggest that water drinking engages sympathetic efferents through a spinal reflex mechanism [8]. The stimulus setting off the response is hypoosmolarity rather than water temperature or gastrointestinal stretch [5], [7], [9]. We identified hepatic spinal afferents in mice detecting physiological shifts in blood osmolality through activation of the transient receptor potential vanilloid cation channel 4 (TRPV4) [10]. Genetic TRPV4 deletion, abolishes the water drinking-induced pressor response [5]. Therefore, we hypothesized that hepatic afferent nerves are involved in the sympathetic activation associated with water drinking and that hepatic denervation attenuates the response. Liver transplant recipients served as hepatic denervation model.

## Methods

The protocol for this trial and supporting CONSORT checklist are available as supporting information; see Checklist S1 and Protocol S1.

### Participants

We included women and men aged 18–60 years who had undergone orthotopic liver transplantation 3–24 months before the study. Kidney transplant recipients who had been transplanted 3–24 months before the study served as immunosuppressive drug matched control group. Patients with psychiatric diseases, alcohol or drug dependence, clinically relevant cardiovascular disease, or transplantation of another organ were excluded. All patients were recruited in the Hannover Medical School transplant clinic.

### Ethics

Written informed consent of the subjects was obtained before study entry. The study has been approved by the institutional review board of Medical School Hannover. Before initiation, the study has been registered on ClinicalTrials.gov (NCT01237431).

### Intervention

All tests were conducted in the morning hours after an overnight fast. Patients did not drink for at least 90 minutes before the study. We asked patients to empty the bladder before the test. Throughout the test, patients remained in a comfortable seated position with both legs elevated. We measured respiration and electrocardiogram continuously (Cardioscreen, Medis GmbH, Ilmenau, Germany). We also determined beat-by-beat blood pressure (Finapres, Ohmeda, Englewood, CA, U.S.A.) and brachial arterial blood pressure (Dinamap, Critikon, Tampa, FL, U.S.A.). Furthermore, we inserted a catheter in an antecubital vein for blood sampling.

After a resting period of at least 15 minutes, we started the baseline recording for 15 minutes. Then, patients ingested 500 ml tap water at room temperature in less than 5 minutes. We continued the recordings for another 60 minutes. Venous blood samples were obtained 15 and 0 minutes before and 15, 30, 40, and 60 minutes after water ingestion.

### Objectives

We hypothesized that sympathetic activation associated with water drinking is attenuated in liver compared to kidney transplant recipients.

### Primary endpoint

The prespecified primary endpoint of the study was the difference between the averaged norepinephrine concentration 30–40 minutes after water ingestion and the averaged baseline norepinephrine concentration (−15 and 0 minutes). [Protocol S1]

### Sample size calculation and statistical analysis

In previous experiments in older healthy subjects (n = 7), venous plasma norepinephrine increased by 0,51 nmol/L (86 pg/ml) with a standard deviation of 0,17 nmol/L (29 pg/ml) thirty minutes after water ingestion. We hypothesized that liver afferent input explains at least 75% of the sympathetic activation with water drinking. Thus, liver transplant recipients would show a 75% reduction in the plasma norepinephrine increase after water ingestion compared with kidney transplant recipients. As estimated before recruitment inclusion of 20 patients in each group provided a more than 80% statistical power to show such differences (two sided, alpha = 5%). To provide a more comprehensive measure of the blood pressure response to water drinking, we calculated the area under the curve of the change in systolic finger blood pressure between 10 and 60 minutes after water drinking. All data are expressed as mean±SEM. Intraindividual differences were compared by the paired t-test. ANOVA testing for repeated measures was used for multiple comparisons. A value for p<0.05 was considered significant.

### Catecholamine analysis

Venous blood samples for catecholamine analysis were collected in EGTA-tubes (Kabevette®, Kabe Labortechnik GmbH, Nümbrecht-Elsenroth, Germany) and processed immediately in a refrigerated centrifuge. The plasma was stored at −80°C until analysis. Plasma epinephrine and norepinephrine were assayed by high-pressure liquid chromatography with electrochemical (amperometric) detection [11].

### Data acquisition and analysis

ECG, finger blood pressure and thoracic impedance signals were analog to digital converted at 500 Hz using the Windaq Pro+ software (Dataq Instruments Inc., USA). R-R intervals (time between subsequent R waves in the ECG), blood pressure, and bioimpedance signals were analyzed off-line using a program written by André Diedrich based on PV-wave software (Visual Numerics Inc., USA). Cardiac stroke volume was calculated according to Sramek's formula [12]. Cardiac output was calculated as product of stroke volume and heart rate. Systemic vascular resistance was calculated as mean finger blood pressure divided by cardiac output. Standard indices of heart rate variability and blood pressure variability were determined by spectral analysis from artifact-free recordings of ECG and finger blood pressure, respectively, in the high (0.15–0.4 Hz) and low frequency (0.04–0.15 Hz) bands [13]. Spontaneous baroreflex sensitivity (BRS) was calculated as the slope of the linear regression lines between the SBP and the subsequent R-R intervals (within the same or the next heart beat) values using the sequence technique. Sequences with at least three intervals, 0.5 mmHg blood pressure changes, and 5 msec R-R interval changes were analyzed only if the correlation coefficients were >0.85 [14].

## Results

### Patients characteristics

**Figure 1:**
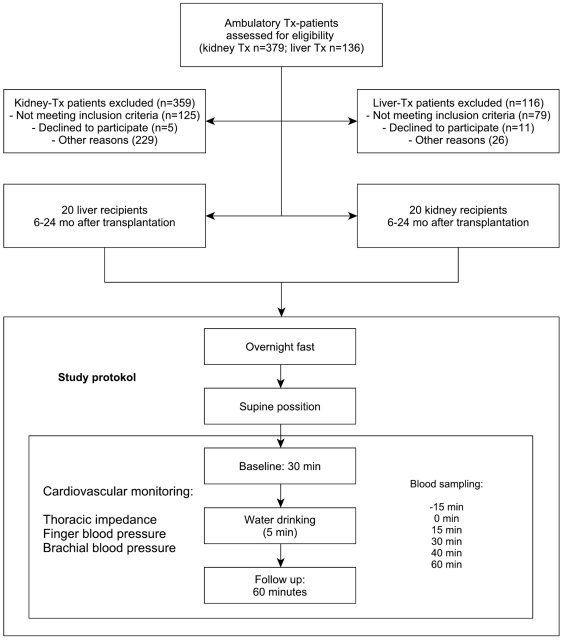
Enrollment and Study-protocol.

Figure 1 shows a flow diagram illustrating the disposition of patients and the study protocol of the trial. Twenty liver transplant recipients and 20 kidney transplant recipients completed the study. Anthropometric data and clinical characteristics are given in table 1. Both groups were well matched for age, gender, BMI, and immunosuppressive drugs. Blood pressure was similar in both groups (p = 0.7). However, more kidney transplant recipients were on antihypertensive medications, particularly beta-adrenoreceptor blockers. Liver and kidney transplant recipients had been transplanted 1.2±0.1 and 0.8±0.1 years before the study, respectively.

**Table 1:** Patients characteristics.

	liver recipients	kidney recipients
Total (male,female)	20 (10/10)	20(10/10)
Body mass [kg]	73.4±5.4	80.9±3.7
Height [m]	1.72±0.0	1.73±0.0
BMI [kg/m^2^]	24.6±1.6	27.2±1.2
Age [years]	44±2.6	42.6±2.6
Time since TX [years]	1.2±0.1	0.8±0.1
Total number of Medication	7.85 p.c.	9.5 p.c.
Systolic Blood Pressure	124±3.9 mmHg	122±4.2 mmHg
Plasma Norepinephrine	1.52±0.14 nmol/L	1.52±0.15 nmol/L
Total number of Immunosuppressant drugs	2.9 p.c.	3 p.c.
Total number of Antihypertensive drugs	0.7 p.c.	2.75 p.c.
Total number of other Medications (p.c.)	4.3 p.c.	3.8 p.c.
Calcineurininhibitor	95%	90%
Mycophenolate	90%	95%
Cortisone	95%	100%
mTor Inhibitior	5%	10%
Azathioprine	5%	5%
Beta blockers	25%	85%

### Plasma catecholamines

**Figure 2:**
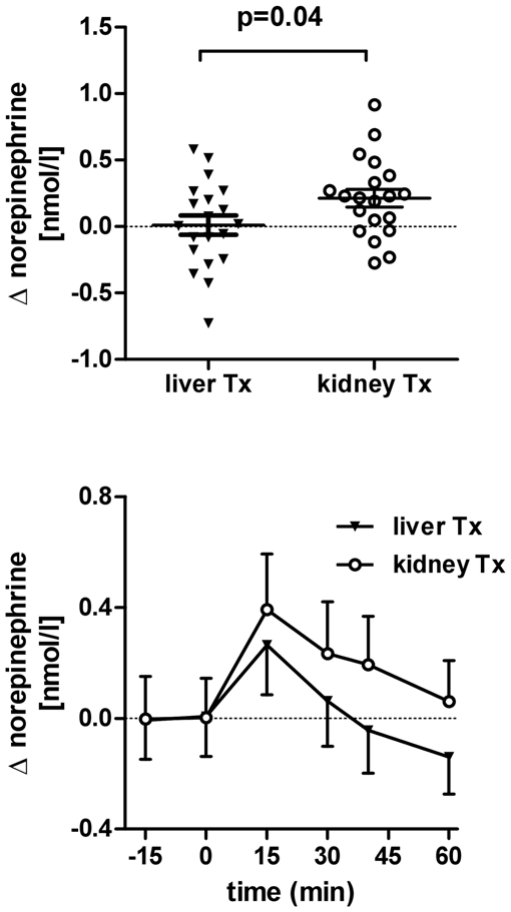
Top – Individual change in venous plasma norepinephrine in liver (liver Tx) and in kidney transplant (kidney Tx) patients. The change in plasma norepinephrine was calculated as the difference in averaged plasma norepinephrine 30–40 minutes after water drinking and averaged baseline norepinephrine. Bottom – Change in venous plasma norepinephrine over time. Patients started drinking water at 0 minutes.

Baseline catecholamines did not differ between groups (p = 0.98, table 1). Figure 2 shows individual changes in averaged venous plasma norepinephrine concentrations 30–40 minutes after water drinking, the prespecified primary endpoint of the study. Plasma norepinephrine 30–40 minutes after water drinking changed 0.21±0.07 nmol/L in kidney transplant recipients and 0.01±0.07 nmol/L in liver transplant recipients (p<0.05). Intraindividual plasma norepinephrine variability at rest calculated from −15 and 0 minute values was −0.009 nmol/L with a confidence interval of −0.073 to 0.056. Venous plasma epinephrine changed −0.01±0.00 nmol/L in liver and −0.01±0.01 nmol/l in kidney transplant recipients (p = 0.54 between groups, not shown).

### Hemodynamic response to water ingestion

**Figure 3:**
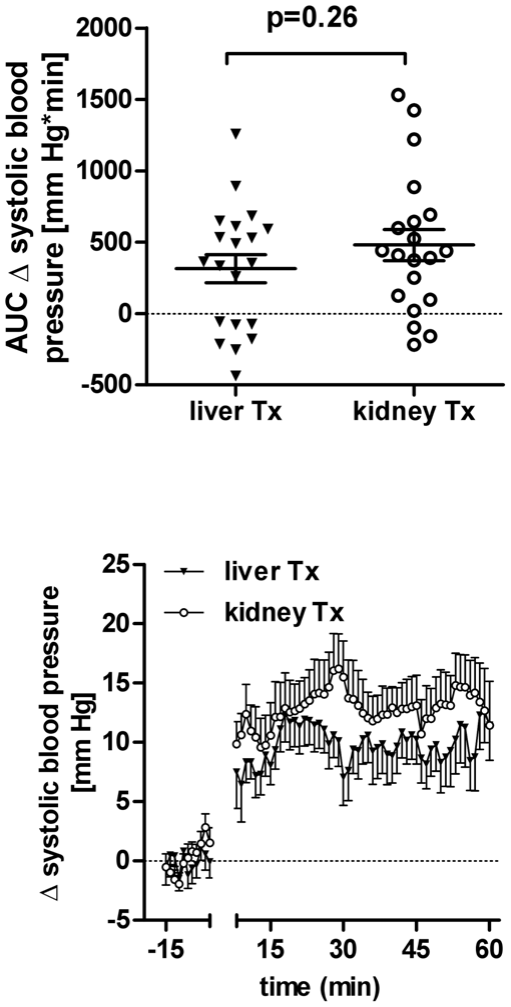
Top – Individual area under the curve of the change in systolic blood pressure between 10 and 60 minutes after water drinking in liver (liver Tx) and in kidney transplant (kidney Tx) patients. Bottom – Change in systolic blood pressure over time. Patients started drinking water at 0 minutes.

**Figure 4:**
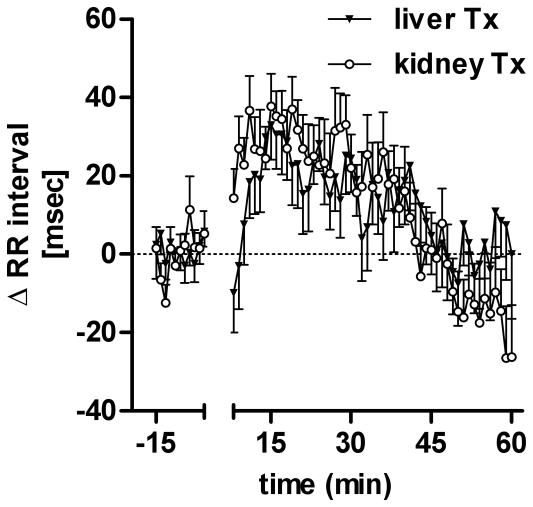
Change in RR interval over time in liver (liver Tx) and in kidney transplant (kidney Tx) patients. Patients started drinking water at 0 minutes.

Figure 3 illustrates systolic finger blood pressure (FBP) changes over time after water ingestion and R-R-Interval time course is shown in Figure 4. Presented are beat-to-beat values averaged for 60 seconds. We excluded the time patients required for drinking water. Systolic finger blood pressure increased 9.3±2.1 mmHg in liver transplant (p<0.001) and 13±2.3 mmHg (p<0.001) in kidney transplant recipients (p = 0.23 between groups 30–40 minutes after water drinking). Individual data on the finger blood pressure response to water quantified as area under the curve are shown in Figure 3 (top). The area under the curve for change in finger blood pressure was 313±97.6 mmHg*min in liver and 480±109 mmHg*min in kidney transplanted patients (p = 0.26 between groups). With water drinking, heart rate decreased 2.01±0.64 bpm in liver and 1.01±0.66 bpm in kidney transplant recipients (p = 0.28 between groups).

**Figure 5:**
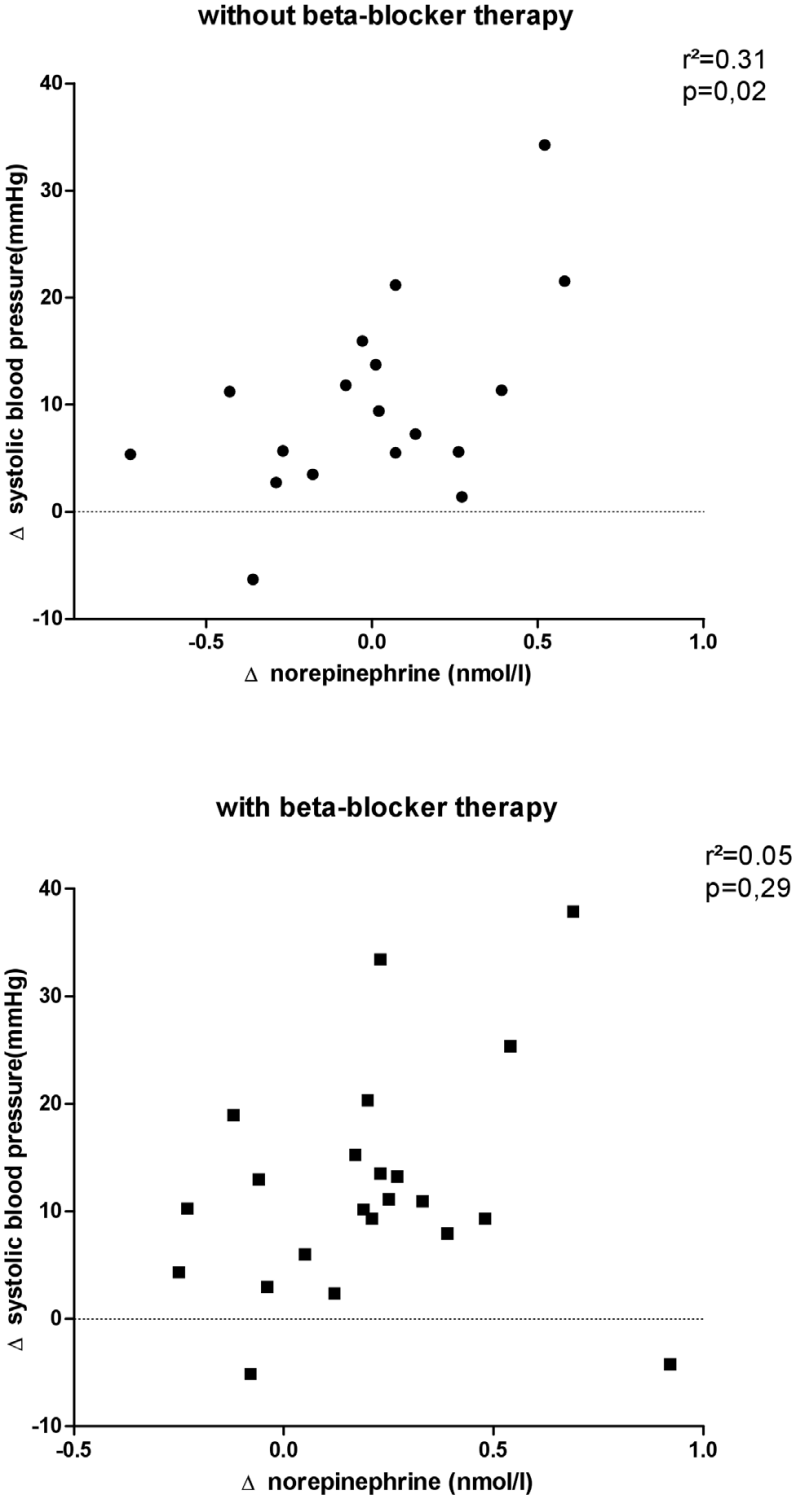
Correlation between changes in plasma norepinephrine and change in systolic blood pressure in patients with (top) and without concomitant beta-blocker therapy (bottom). We averaged two measurements obtained 30 and 40 minutes after water drinking.

Treatment with beta-adrenoreceptor blockers could attenuate the sympathetically mediated pressor response to water drinking, as shown in case of attenuated metabolic changes after water drinking [6]. Therefore, we plotted changes in finger blood pressure over changes in venous plasma norepinephrine concentration 30–40 minutes after water ingestion separately for patients with and without beta-adrenoreceptor blocker treatment. We observed a positive correlation between change in systolic blood pressure and change in norepinephrine in patients without beta-adrenoreceptor blockade (n = 18, r^2^ = 0.31, p<0.05) but not in patients on beta-adrenoreceptor blockade (n = 22, r^2^ = 0.05, p = 0.29) as shown in Figure 5.

30–40 minutes after water drinking, cardiac stroke volume had changed −5.3±2.1 ml in liver and 1.2±2.9 ml in kidney transplant recipients (p = 0.7). At this time point, cardiac output had changed −0.55±0.16 l/min in liver and 0.02±0.18 l/min in kidney transplant recipients (p<0.05). Total peripheral resistance 30–40 minutes after water drinking had changed 185±92 dyne*s*cm^−5^ in liver and 222±69 dyne*s*cm^−5^ in kidney transplant recipients (p = 0.75 between groups).

## Discussion

The main finding of our study is that in liver transplant recipients plasma norepinephrine increases after water drinking less as compared to the response in kidney transplant recipients. Remarkably, both groups showed a smaller increase in plasma norepinephrine than autonomic failure patients and healthy young or older control subjects in previous studies [1], [2], [4]. The observation may suggest that hepatic afferent nerves are involved in the sympathetic activation associated with water drinking. The pressor response to water drinking was more pronounced in kidney transplant recipients. However, the difference between groups was not statistically significant. Compared with liver transplant recipients, more kidney transplant recipients were treated with beta-adrenoreceptor blockers, which may have attenuated the pressor response to water drinking-induced sympathetic activation.

We studied liver transplant recipients as a human liver denervation model. An earlier study applied PGP 9.5 and anti-S-100 immunostaining to identify nerve fibers in liver samples obtained from the diseased liver and up to four years after transplantation from the donor liver [15]. Early after transplantation, portal and parenchymal nerve fibers had disappeared. Thereafter, a minority of biopsies showed innervation of few small portal tracts [15]. Another study assessed hepatic sympathetic innervation 1, 3, 6, 12, and 30 months after transplantation [16]. Norepinephrine concentration was profoundly reduced compared with samples from non-transplanted patients. Tissue norepinephrine did not recover over time. Finally, abnormalities in glucose metabolism in transplant recipients that have been attributed to liver denervation did not recover 28 months following transplantation [17]. Together, these findings suggest that liver transplant recipients studied 3–24 months after transplantation provide a valid human liver denervation model. We decided to study kidney transplant recipients as control group because in our institution, both patient groups are treated with similar immunosuppressive agents.

We observed a biphasic sympathetic and hemodynamic response to water drinking in kidney transplant recipients. The initial norepinephrine response was virtually identical in both groups suggesting that sympathetic efferent neurons can be engaged by central nervous stimuli. However, the second phase of the change in plasma norepinephrine after water drinking differed markedly between liver and kidney transplant recipients. With water drinking, plasma norepinephrine concentrations increased in kidney transplant recipients. Liver transplant recipients hardly responded to water drinking. Thus, loss of liver innervation is associated with reduced sympathetic activation following water drinking.

The fact that water drinking elicited a smaller increase in plasma norepinephrine in our control group of kidney transplant recipients compared to earlier results in older adults without immunosuppressant drugs may suggest that immunosuppressive medications, particularly calcineurin inhibitors, may have led to structural or functional alterations of postganglionic adrenergic neurons. Indeed, neurotoxicity is a common adverse reaction to calcineurin inhibitor treatment [18], [19]. Another, less likely, explanation for the attenuated water drinking-induced norepinephrine release in our kidney transplanted patients is that renal afferent nerves may also contribute to the sympathetic activation after water drinking. Indeed, renal afferent nerves may drive an increase in efferent sympathetic activity in patients with renal disease. Removal of a diseased kidney can lower sympathetic activity and blood pressure in patients with end stage renal disease [20]. Moreover, chemical stimulation of renal afferent nerves through phenol injection raises efferent sympathetic activity and blood pressure in animal models [21]. Yet, a large proportion of osmosensitive spinal afferents appear to originate in the liver rather than the kidneys at least in mice [10]. Finally, renal sympathetic efferents, which are severed in renal transplant recipients, may contribute to the change in systemic norepinephrine concentration with water drinking [22].

The primary goal of our study was to assess influences of hepatic denervation on water drinking induced sympathetic activation. Even though the plasma norepinephrine response to water drinking was substantially reduced in liver compared with kidney transplant recipients, the pressor response did not differ significantly between groups. The pressor response to water drinking-induced sympathetic activation is exacerbated in patients and in animals with impaired baroreflex function [1], [4], [5], [23]. Baroreflex sensitivity determined by cross spectral analysis and by the sequence method was similar in liver and in kidney transplant recipients. Therefore, it is unlikely that the surprisingly small difference in the pressor response to water drinking between liver and kidney transplant recipients is explained by a group difference in baroreflex function. The fact that renal transplant recipients received more beta-adrenoreceptor antagonists is a more likely explanation for the dissociation between norepinephrine release and pressor response. Antiadrenergic medications abolish the pressor response to water drinking, both, in animals and in autonomic failure patients [1], [5]. Furthermore, beta-adrenoreceptor blockade attenuates the increase in metabolic rate with water drinking [6]. In the present study, the positive correlation between changes in plasma norepinephrine and changes in systolic blood pressure was abolished in beta-adrenoreceptor blocker treated patients. We did not find it ethically acceptable to wash out antihypertensive medications in our study given the markedly increased cardiovascular risk in transplant patients.

### Perspectives

Our study suggests that hepatic afferent nerves contribute to water drinking-induced sympathetic activation. Animal studies suggested that water drinking induces hypoosmolarity in the portal vein, thus, activating TRPV4 channels on hepatic spinal afferents [5], [10]. The afferent stimulus may then activate efferent sympathetic nerves at the spinal level [8]. The increase in sympathetic tone raises blood pressure in animals and in human subjects with impaired baroreflex function, the so called osmopressor response [5], [24]. We believe our study has scientific and clinical implications. First, our study suggests that afferent nerves originating in the liver affect human autonomic regulation. Second, water drinking improves postprandial and orthostatic hypotension in patients with severe autonomic failure [25]. Water drinking also prevents (pre)syncope in otherwise healthy subjects by severe orthostatic stress and during blood donations as well as in neurally mediated syncope patients [26]–[29]. Thus, our findings may help develop new treatments for these conditions. Finally, water drinking-induced sympathetic activation raises metabolic rate. Manipulation of osmosensitive hepatic afferents may have utility in the prevention of weight gain. Indeed, increased water ingestion improves weight loss [30], [31]. The beneficial response is at least in part independent of caloric intake or physical activity.

## Supporting Information

Protocol S1 **Original trial protocol (German).** Date of approval by the local ethics committee 3 September 2009.
(PDF)

Checklist S1 **CONSORT Checklist.**
(DOC)
