# Supplementary material for: Liver Afferents Contribute to Water Drinking-Induced Sympathetic Activation in Human Subjects: A Clinical Trial
Source: PLoS One. 2011 Oct 7;6(10):e25898. doi: 10.1371/journal.pone.0025898 (PMC3189227; doi:10.1371/journal.pone.0025898)
Supplement: Protocol S1 — Original trial protocol (German). Date of approval by the local ethics committee 3 September 2009. (PDF) [file pone.0025898.s001.pdf]

Institut für Klinische Pharmakologie

Medizinische Hochschule Hannover

(Direktor: Prof. Dr. med. Jens Jordan)

Studienprotokoll

Bedeutung der Leberinnervation für den Osmopressor Reflex beim Menschen (HEP-Reflex- Studie)

- Humanexperiment, erfüllt nicht die Kriterien einer klinischen Prüfung nach AMG -

Dr. med . Stefan Engeli (Studienleiter)

Protokollversion 1 vom 18. August 2009

1 Zusammenfassung

Bei Patienten mit autonomer Dysfunktion aktiviert Trinken von Wasser den Sympathikus und führt so zu einem starken Blutdruckanstieg (1). Bei Gesunden steigt der Blutdruck nicht oder nur geringfügig, der Ruheenergieumsatz nimmt jedoch um > 20% zu. Diese Wirkungen halten für mehr als eine Stunde an (1-3). Sowohl isoosmolare Lösungen als auch die Magendehnung wurden als Stimuli der Sympathikusaktivierung ausgeschlossen (4). Untersuchungen am Menschen zeigen, dass die Sympathikusaktivierung nach Wassertrinken durch einen spinalen Reflex vermittelt wird (5). Anhand von Tiermodellen wurde gezeigt, dass dieser spinale Reflex vermutlich durch hepatische Osmorezeptoren ausgelöst wird (6-8).

In der vorliegenden Studie wollen wir die sympathische Reaktion gemessen am Anstieg der venösen Plasmakonzentration von Noradrenalin nach dem Trinken von 500 ml Wasser bei Patienten nach Lebertransplantation und bei einer Kontrollgruppe von Nierentransplantierten untersuchen. Bei Lebertransplantierten sind die hepatischen Afferenzen unterbrochen, während sie bei Nierentransplantierten intakt sind. Beide Gruppen werden hinsichtlich Alter, Geschlecht, Begleiterkrankungen sowie immunsuppresiver Medikation gematcht. Auf diese Weise wollen wir ermitteln, ob die reflexvermittelte Sympathikusaktivierung beim Menschen auch dann hervorgerufen wird, wenn spinale Afferenzen der Leber unterbrochen sind. Außerdem werden wir untersuchen, ob die Unterbrechung der Leberafferenzen bei Lebertransplantierten mit einer Störung der Osmoregulation verbunden ist.

2 Verantwortlichkeiten

Institut für Klinische Pharmakologie

Dr. med. Stefan Engeli (532 2817; engeli.stefan@mh-hannover.de)

- Studienleiter
- Entwurf des Studienprotokolls, Antrag bei der Ethikkommission
- Patientenaufklärung
- Datenmanagement und Datenanalyse

Prof. Dr. med. Jens Jordan (532 2821; jordan.jens@mh-hannover.de)

- Entwurf des Studienprotokolls, Antrag bei der Ethikkommission
- Datenanalyse

Marcus May, Arzt (532 2722; May.Marcus@mh-hannover.de)

- Studienprotokoll, Antrag bei der Ethikkommission
- Patientenaufklärung
- alle Untersuchungen, Datenmanagement

Claudia Kunze (532 9337; kunze.claudia@mh-hannover.de)

- Study Nurse

Klinik für Gastroenterologie, Hepatologie und Endokrinologie

PD Dr. med. Heiner Wedemeyer (532-6814; wedemeyer.heiner@mh-hannover.de)

3 Wissenschaftlicher Hintergrund

Das Trinken von Wasser hat erhebliche Auswirkungen auf das sympathische Nervensystem, die therapeutisch nutzbar gemacht werden können (1). Bei Patienten mit autonomer Dysfunktion wird durch das Trinken von normalem Leitungswasser ein starker Blutdruckanstieg ausgelöst. Bei älteren gesunden Menschen steigt der Blutdruck nur wenig an. Bei jüngeren Menschen ändert sich der Blutdruck nicht, die Herzfrequenz nimmt jedoch deutlich ab (9-10). Diese Reaktion wird durch eine Aktivierung des Sympathischen Nervensystems erklärt (11). Tatsächlich steigen nach Wassertrinken sowohl die Noradrenalinkonzentration im Plasma als auch die mikroneurographisch abgeleitete sympathische Nervenaktivität an und der Blutdruckanstieg wird durch pharmakologische Ganglienblockade verhindert (2,3,11). Weiterhin wurde ein Anstieg des Grundenergieumsatzes um bis zu 30% für die Dauer von etwa 60 Minuten nach dem Trinken von 500 ml Wasser beobachtet (2,3), der durch Adrenorezeptorblockade verhindert wurde (2).

Magendehnung, Wassertemperatur sowie der durch das Wasser hervorgerufene Volumeneffekt konnten als Stimuli weitestgehend ausgeschlossen werden (3,11). Ebenso wurden keine Veränderungen der Plasma-Renin und Vasopressin-Konzentrationen nachgewiesen (11). Es liegen Hinweise dafür vor, dass die Reaktion auf Wasser durch einen spinalen Reflex hervorgerufen wird, da der Blutdruckanstieg auch bei tetraplegischen Patienten auslösbar war (4). Da die kardiovaskulären Reaktionen auf Wassertrinken aber nicht durch isotonische Lösungen hervorgerufen werden, geht man von einer osmotisch vermittelten Reaktion aus (8,12). Die hierfür verantwortlichen Osmorezeptoren werden in der Leber vermutet (5-7).

Aufgrund der geschilderten Datenlage ist als Ursache der sympathischen Reaktion auf das Trinken von 500 ml Leitungswasser ein spinaler Reflex anzunehmen, der durch hepatische Osmorezeptoren aktiviert wird. Die Hypothese der vorliegenden Studie ist, dass die Reaktion des sympathischen Nervensystems auf Wassertrinken bei Patienten nach Lebertransplantation nicht mehr auftritt, weil durch die Denervierung des Transplantats hepatische spinalen Reflexbögen unterbrochen sind.

4 Durchführung der Studie

Zunächst werden unter Mithilfe der Transplantationsambulanz der MHH geeignete Patienten nach Lebertransplantation und nach Nierentransplantation ausgewählt.

Wenn grundsätzliche Bereitschaft besteht, erfolgt die Aufklärung über Sinn, Ziele und Risiken der Studienteilnahme. Zudem werden Informationen zum Datenschutz mitgeteilt. Nach Beantwortung aller Fragen des Patienten werden die schriftliche Patienteninformation und Einwilligungserklärung ausgehändigt. Die Einwilligungserklärung wird vom Patienten und vom Studienarzt unterschrieben und handschriftlich datiert. Der Patient erhält ein Exemplar der Patienteninformation und Einwilligungserklärung zu seiner Verfügung.

Nach Einwilligung werden folgende Daten erhoben: Geschlecht, Alter, Körpergewicht und Körpergröße, Begleiterkrankungen, Begleitmedikation, Raucherstatus.

Am Untersuchungstag kommen die Studienteilnehmer nüchtern um 8:00 in das Studienzentrum. Der Ablauf ist dann wie folgt:

- Legen eines peripheren venösen Katheters
- Anlage einer Oberarm-Blutdruckmanschette, EKG, Finapress-Fingermanschette
- 30 min Ruhephase
- Regelmäßige Blutdruckmessung durch Oberarmmanschette alle 5 Minuten
- Blutabnahmen bei Minute -30, -15 und 0 (je 7 ml EDTA-Röhrchen und Katecholamin-Röhrchen)
- Kontinuierliche Blutdruckmessung von Minute -10 bis 60
- Trinken von 500 ml Wasser innerhalb von 5 min
- Blutabnahmen bei Minute 15, 30, 40 und 60 (je 7 ml EDTA-Röhrchen und Katecholamin-Röhrchen)

Die Blutdruckmessung wird mittels automatisierter oszillometrischer Messung durchgeführt (Dinamap) sowie durch nichtinvasive kontinuierliche Blutdruckmessung am Finger (Finapress).

5 Risiken für die Studienteilnehmer; Versicherung; Aufwandsentschädigung

Die Teilnahme an der vorliegenden Studie birgt nur minimale Risiken für die Studienteilnehmer. Durch den venösen Katheter und die damit verbundene Punktionen können in seltenen Fällen Blutungen, Infektionen oder Verletzungen von Hautnerven entstehen. In ca. 15% wird ein subkutanes Hämatom entstehen.
Der Studiencharakter bedingt, dass die Studie weder unter die Regeln des Arzneimittel- noch des Medizinproduktegesetzes fällt. Damit reicht die Betriebshaftpflichtversicherung der Medizinischen Hochschule Hannover aus, eine zusätzliche Probandenversicherung ist nicht notwendig.

Eine Aufwandsentschädigung wird nicht gezahlt.

6 Auswahl der Probanden

6.1 Einschlusskriterien

- Patienten drei bis 24 Monate nach Nierentransplantation sowie Patienten drei bis 24 Monate nach Lebertransplantation
- Männer und Frauen im Alter von 18 bis 60 Jahren
- Fähigkeit zum Verständnis der mündlichen und schriftlichen Aufklärung und Abgabe der unterzeichneten Einverständniserklärung

6.2 Ausschlusskriterien

- Schwangerschaft und Stillzeit
- Transplantation eines anderen Organs als Leber oder Niere
- chronische oder akute Herz- und Gefäßerkrankungen
- bekannter Alkohol- und/oder Drogenmissbrauch
- psychiatrische Erkrankungen akut oder in der Vorgeschichte (Depression, Schizophrenie, Essstörungen, Suchterkrankungen

7 Datenverarbeitung und Statistik

7.1 Datenverarbeitung

Die erhobenen Daten werden in Papierform gelagert und in eine SPSS-Tabelle zur statistischen Analyse übertragen. Die Laborwerte werden in der Regel in EXCEL-Tabellen berechnet und von dort in die SPSS-Tabelle übertragen. Die

Datenschutzbestimmungen des Landes Niedersachsen werden beachtet. Die Aufbewahrung der Studienunterlagen erfolgt für 10 Jahre.

Alle erhobenen Daten werden unter einem Pseudonym gespeichert und verarbeitet. Das Pseudonym besteht aus einer studienspezifischen Nummer, die keine personenbezogenen Hinweise enthält. Die Publikation der Daten in einem internationalen Peer Review Journal wird angestrebt. Auch hier werden nur pseudonymisierte Werte oder Gruppenmittelwerte verwendet, keine individuell zuzuordnenden Daten. Eine Weitergabe der Daten an Dritte wird nicht vorgenommen.

7.2 Hypothese

Wir vermuten, dass Wassertrinken hepatische Osmorezeptoren aktiviert und so über hepatische spinale Afferenzen eine Sympathikusaktivierung auslöst. Bei Lebertransplantierten erwarten wir eine deutlich reduzierte Sympathikusaktivierung, weil bei diesen Patienten die Leber denerviert ist.

7.3 Primärer Studienendpunkt

Ermittlung eines Unterschiedes im Noradrenalinanstieg nach dem Trinken von Wasser bei Lebertransplantierten im Vergleich zu Nierentransplantierten. Dabei wird für jeden Patienten die Differenz zwischen dem Mittelwerte der Noradrenalinkonzentration nach Wassertrinken (gemittelt 30 und 40 Minuten) und dem Wert vor Wassertrinken (gemittelt -30, -15, 0 Minuten) berechnet. Anhand von Literaturdaten und eigenen Untersuchungen erwarten wir die maximale Sympathikusaktivierung 30-40 Minuten nach Wassertrinken.

7.4 Sekundärer Studienendpunkt

Zeitverlauf der Noradrenalinwerte nach Wassertrinken

Änderung von Herzfrequenz, Blutdruck, Osmolarität im Zeitverlauf

7.5 Fallzahlberechnung

Für die bisherigen Studien zur Untersuchung der Wirkung von Wasser wurden Fallzahlen von etwa 10-15 Probanden für jede Gruppe ausgewählt. Durch diese Anzahl an Studienteilnehmern konnten Auswirkungen bei Gesunden, Älteren und Patienten mit autonomen Störungen nachgewiesen werden (2, 9).

Für die Fallzahlberechnung wurde davon ausgegangen, dass ein sympathischer Reflex ausgehend von der Leber mindestens zu 3/4 an der Reaktion auf das Trinken von Wasser beteiligt ist. Aufgrund der Morbidität und Voralterung der zu untersuchenden transplantierten Patienten, wird eine ähnliche Reaktion erwartet wie zuvor bei den älteren Probanden.

Fallzahlberechnung (Sample Size Calculator DSS (www.dssresearch.com)):

Literatur (11):

Ältere Pat.:	Noradrenalin			
Pat. Nr.	0 min	30 min	Veränderung	Ohne Pat 6
1	338	477	139	139
2	599	698	99	99
3	266	326	60	60
4	246	319	73	73
5	264	350	86	86
6	351	768	417	
7	428	521	93	93
8	227	278	51	51
		Durchschnitt:	127,25	85,86
		SD:	120,15	29,15

Berechnung aktuelle Studie:

Sample size calculator DSS/ / two sided / alpha=0.05

		Mittelwert	SD	n	Power (%)
Wirkung nur hepatisch	NTX	127	120	11	92
	LTX	0			
Wirkung 3/4 hepatisch	NTX	127	120	24	72
	LTX	30			
Ausschluss von Pat.6	NTX	86	29	15	100
Wirkung 3/4 hepatisch	LTX	20			

Aufgrund der Literaturergebnisse und der Annahme, dass etwa 75% der Reaktion auf das Trinken von Wasser durch Osmorezeptoren in der Leber sowie einen spinalen Reflex ausgelöst wird, sollte durch eine Stichprobengröße von jeweils 20 Probanden ausreichen. Außerdem werden wir die Erfolgsaussichten der Studie dadurch erhöhen, dass wir im Vergleich zu Voruntersuchungen die Variabilität der Noradrenalinwerte durch Standardisierung der Abnahmebedingungen, Verwendung spezifischerer Assays und Wiederholungsmessungen reduzieren.

7.6 Auswertung primärer und sekundärer Zielvariablen:

Die Nullhypothese: „Es besteht kein Unterschied in den Gruppen hinsichtlich der Änderung der venösen Noradrenalinkonzentration nach Wassertrinken“ wird mittels

zweiseitigem T-Test unabhäniger Stichproben mit einem Signifikanzniveau alpha= 0,05 auf Signifikanz geprüft. Die Zeitverläufe der primären und sekundären Zielvariablen werden mittels Varianzanalyse untersucht (Two-Way-ANOVA).

Literatur

1. Jordan J. Acute effect of water on blood pressure. What do we know? Clin Auton Res 2002 Aug;12(4):250-5.

2. Boschmann M, Steiniger J, Hille U, Tank J, Adams F, Sharma AM, et al. Water-induced thermogenesis. J Clin Endocrinol Metab 2003 Dec;88(12):6015-9.

3. Boschmann M, Steiniger J, Franke G, Birkenfeld AL, Luft FC, Jordan J. Water drinking induces thermogenesis through osmosensitive mechanisms. J Clin Endocrinol Metab 2007 Aug;92(8):3334-7.

4. Tank J, Schroeder C, Stoffels M, Diedrich A, Sharma AM, Luft FC, et al. Pressor effect of water drinking in tetraplegic patients may be a spinal reflex. Hypertension 2003 Jun;41(6):1234-9.

5. Markworth S, Lechner SG, Frahm S, Imai M, Suzuki M, Ibanez-Tallon I, et al. Molecular and cellular characterization of peripheral osmoreceptors. 2009.

6. McHugh J, Keller NR, Appalsamy M, Raj SR, Diedrich A, Liedtke W, et al. An osmopressor mechanism linked to TRPV4 elicits robust blood pressure elevation. 2009.

7. Haberich FJ. Osmoreception in the portal circulation. Fed Proc 1968 Sep-Oct;27(5):1137-41.

8. Lipp A, Tank J, Franke G, Arnold G, Luft FC, Jordan J. Osmosensitive mechanisms contribute to the water drinking-induced pressor response in humans. Neurology 2005 Sep 27;65(6):905-7.

9. Jordan J, Shannon JR, Grogan E, Biaggioni I, Robertson D. A potent pressor response elicited by drinking water. Lancet 1999 Feb 27;353(9154):723.

10. Young TM, Mathias CJ. The effects of water ingestion on orthostatic hypotension in two groups of chronic autonomic failure: multiple system atrophy and pure autonomic failure. J Neurol Neurosurg Psychiatry 2004 Dec;75(12):1737-41.

11. Jordan J, Shannon JR, Black BK, Ali Y, Farley M, Costa F, et al. The pressor response to water drinking in humans : a sympathetic reflex? Circulation 2000 Feb 8;101(5):504-9.

12. Brown CM, Barberini L, Dulloo AG, Montani JP. Cardiovascular responses to water drinking: does osmolality play a role? Am J Physiol Regul Integr Comp Physiol 2005 Dec;289(6):R1687-92.

13. Jordan J, Shannon JR, Diedrich A, Black B, Robertson D, Biaggioni I. Water potentiates the pressor effect of ephedra alkaloids. Circulation 2004 Apr 20;109(15):1823-5.
